# News and Events of the Week

**Published:** 1909-12-18

**Authors:** 


### News and Events of the Week.

In response to an advertisement inserted in two New York papers recently, offering a substantial reward to a young, healthy man willing to be a donor in a transfusion operation, sixty applicants appeared.

Mr. George Crocker, the son of a Californian railway financier, who left an estate of £6,000,000, has given to Columbia University a fund estimated at £300,000 for the investigation of cancer. Mr. Crocker and his wife were both victims of that disease.

The Army Council has approved of the renewal for a period of three years of the appointments of the following gentlemen as members of the Army Medical Advisory Board: Dr. J. Rose Bradford, F.R.S., Sir C. Cameron, C.B., Dr. L. C. Parkes, Dr. M. S. Pembrey, and Sir F. Treves.

The following appointments and elections have been made at a meeting of the Council of the Royal College of Surgeons: Mr. J. F. Colyer, M.R.C.S., L.D.S.Eng., honorary curator of the Odontological Collection in the museum. Mr. H. F. Waterhouse, F.R.C.S., surgeon to Charing Cross Hospital, was elected a member of the Court of Examiners. Dr. G. F. Blacker, obstetric physician at University College Hospital, was elected an examiner in midwifery under the Conjoint Examining Board. Mr. R. J. Godlee was elected a representative of the College on the Senate of the University of London, and Mr. A. P. Gould was elected a member of the Committee of the Imperial Cancer Research Fund.

An anonymous donor has offered a prize of £20,000 for the discovery of an absolute cure for tuberculosis. Yale University is the custodian of this prize, and the physicians attached to the Yale Medical School are to act as trustees. The trustees have invited many well-known physicians to become members of an advisory board whose duty it will be to pass on the merits of cures submitted. The prize is open to any scientist or physician the world over. The money will be held in trust, it is understood, and the interest from it will go toward investigating any cures the trustees or members of the advisory board hear of, but that are not submitted to them for examination. The advisory board plans to hold four meetings a year, and will hold others if the cures or alleged cures that are submitted warrant it. One of the duties of the advisory board will be the exposure of "fake" consumption cures which may come to their notice.

The result of the examination for visiting doctors to the Paris hospitals places a lady, Mademoiselle Romme, at the head of the list, above all the men competitors.

A serious epidemic of measles has been reported in the Rhymney Valley, South Wales. Eight schools, with an average attendance of 2,150, have been closed, some of them till the New Year.

The Corporation of Glasgow has carried a resolution by which tuberculosis of the lung, or tubercular phthisis, shall be included among the notifiable diseases under the Infectious Diseases (Notification) Act.

Dr. Martha Adams has been awarded one farthing damages in the King's Bench Division of the High Court as the result of her action for alleged libel against *Vanity Fair* in respect of an article written by the Hon. Ernest Pomeroy.

The jubilee of the granting of the Charter by the Royal College of Surgeons, embodying a standard of qualification for the dental profession, was celebrated on December 2 by a dinner to many representatives of the dental profession held in the library of the college.

Sir George M'Crae, Vice-President of the Local Government Board, opened at Edinburgh, on December 3, a sale of work made by the inmates of Craigleith and Craiglockhart Poorhouses. He said that ladies of the Brabazon Employment Society, who gave work to those people who otherwise might be kept in enforced idleness, did a great service. As Vice-President of the Local Government Board, he could say that one of the most difficult duties the Board had to perform was looking after Poor Law administration.

The Guardians of Shillingford recently discussed the cause of a local outbreak of diphtheria, resulting in two fatal cases. An opinion was expressed that the germs of the disease have been conveyed by cats, several of which were known to be unwell in the village. The carcass of one was found on bacteriological examination to be affected with diphtheria. One cat at Shillingford caught the infection from a human patient. A house-to-house visitation was made in the district in search of ailing cats, and the inhabitants were advised to destroy every cat which appeared sick.

